# Large-scale screening of transcription factor–promoter interactions in spruce reveals a transcriptional network involved in vascular development

**DOI:** 10.1093/jxb/eru116

**Published:** 2014-04-08

**Authors:** Isabelle Duval, Denis Lachance, Isabelle Giguère, Claude Bomal, Marie-Josée Morency, Gervais Pelletier, Brian Boyle, John J. MacKay, Armand Séguin

**Affiliations:** ^1^Natural Resources Canada, Canadian Forest Service, Laurentian Forestry Centre, Québec, QC, G1V 4C7, Canada; ^2^Centre d’Étude de la Forêt, Université Laval, Québec, QC, G1V A06, Canada; ^3^Department of Plant Sciences, University of Oxford, Oxford, OX1 2RB, UK

**Keywords:** Conifer, expression pattern, *Picea glauca*, secondary cell wall, somatic embryogenesis, *trans*-activation assay, transcription factor, xylem.

## Abstract

This research aimed to investigate the role of diverse transcription factors (TFs) and to delineate gene regulatory networks directly in conifers at a relatively high-throughput level. The approach integrated sequence analyses, transcript profiling, and development of a conifer-specific activation assay. Transcript accumulation profiles of 102 TFs and potential target genes were clustered to identify groups of coordinately expressed genes. Several different patterns of transcript accumulation were observed by profiling in nine different organs and tissues: 27 genes were preferential to secondary xylem both in stems and roots, and other genes were preferential to phelloderm and periderm or were more ubiquitous. A robust system has been established as a screening approach to define which TFs have the ability to regulate a given promoter *in planta*. *Trans*-activation or repression effects were observed in 30% of TF–candidate gene promoter combinations. As a proof of concept, phylogenetic analysis and expression and *trans*-activation data were used to demonstrate that two spruce NAC-domain proteins most likely play key roles in secondary vascular growth as observed in other plant species. This study tested many TFs from diverse families in a conifer tree species, which broadens the knowledge of promoter–TF interactions in wood development and enables comparisons of gene regulatory networks found in angiosperms and gymnosperms.

## Introduction

Plants produce wood through a process of secondary xylem development and growth with four major steps: initial cell division, cell expansion, cell-wall thickening, and programmed cell death (Plomion *et al.*, 2001). Characteristic structures are thus formed that impart strength to support the plant body and the ability to transport water and nutrients efficiently from the roots to other organs (Miyashima *et al.*, 2013). Secondary vascular growth represents a key adaptation and a major carbon sink in woody perennial plants; however, it is also observed on a smaller scale and studied in herbaceous plants. In *Arabidopsis*, it has been shown that the events of underlying primary and secondary vascular development are under tight regulatory control and involve transcription factors (TFs) from the R2R3-MYB (MYB), basic helix–loop–helix, KNOTTED1-like homeobox (KNOX), homeodomain leucine zipper (HD-Zip), and NAM, ATAF1/2, and CUC2 (NAC)-domain families (Demura and Fukuda, 2007; Wang and Dixon, 2012).

Recent studies have begun to unravel a transcriptional network regulating xylem development and growth. For example, the NAC-domain proteins are plant-specific TFs with several members playing a central regulatory role in xylogenesis, fibre development, and wood formation (Zhong *et al.*, 2010a). Several NAC-domain genes are preferentially expressed in the cambial zone or xylem cells such as vascular elements and in interfascicular fibres (Demura and Fukuda, 2007; Zhong *et al.*, 2010a; Wang *et al.*, 2011; Wang and Dixon, 2012). In *Arabidopsis*, NAC-domain proteins (SND1, NSTs, and VNDs) orchestrate a transcriptional cascade involving MYB regulatory proteins, ultimately activating the transcription of enzymes involved in secondary cell wall (SCW) assembly. A similar cascade involving functional NAC-domain genes called WNDs (Wood-Associated NAC Domain TFs) and MYBs has been described in poplar and eucalyptus (Zhong and Ye, 2009; Zhong *et al.*, 2011).

The vascular tissues of angiosperms and gymnosperms have a number of anatomical and biochemical features in common and also many distinctive properties (Pallardy, 2008). These range from cell types and secondary cell macromolecules (both lignins and hemicelluloses) to intercellular connections and growth responses to stress (e.g. reaction wood). Conifers are a dominant group among the gymnosperms that may serve for comparative developmental studies in addition to being economically and ecologically important. Genomic initiatives in conifers have developed large gene sequence data sets, gene maps, and expression profiles (reviewed by Mackay *et al.*, 2012) and more recently the complete genome sequences of two spruce species (Birol *et al.*, 2013; Nystedt *et al.*, 2013) and a pine (http://www.pinegenome.org/pinerefseq/). These information resources represent a foundation to accelerate the discovery of regulatory mechanisms of secondary growth and wood formation.

A class of transcriptional regulators that has been well studied in conifer vascular development are the R2R3-MYBs. The loblolly pine (*Pinus taeda* L.) genes *PtMYB1* and *PtMYB4* have been shown to be expressed in differentiating secondary xylem and to activate transcription through specific binding to AC motifs from promoters of most of the phenylpropanoid pathway genes (Patzlaff *et al.*, 2003a,b). When overexpressed in *Arabidopsis* and tobacco, these MYBs induced ectopic lignification (Patzlaff *et al.*, 2003a; Newman *et al.*, 2004). Several other R2R3-MYB genes have been characterized in white spruce (*Picea glauca* (Moench) Voss), loblolly pine (Bedon *et al.*, 2007, 2010; Bomal *et al.*, 2008) and in maritime pine (*Pinus pinaster* Ait.; Craven-Bartle *et al.*, 2013). Sequence and phylogenetic analysis showed conservation in gene family structure between conifers and *Arabidopsis*.

Research groups, including the current group, have overexpressed R2R3-MYBs and other TFs in transgenic conifer trees. Constitutive expression of *PtMYB1* or *PtMYB8* in spruce lines resulted in ectopic SCW deposition and increased lignin accumulation (Bomal *et al.*, 2008). Craven-Bartle *et al.* (2013) independently showed that a *Pinus pinaster* homologue of MYB8 is a candidate regulator of phenylpropanoid metabolism and lignin synthesis genes. PtMYB8 is closely related to AtMYB61 and to AtMYB46, which are downstream of NAC-domain regulators (Zhong *et al.*, 2010a), suggesting that PtMYB8 may be part of a transcriptional network controlling SCW deposition in conifers (Bomal *et al.*, 2008).

Nevertheless, the production of stably expressing conifer lines takes several months, and selection of lines with stable TF overexpression could be difficult to obtain due to phytotoxic side effects or other pleiotropic effects. This group has developed a rapid transient transformation system for conifers to circumvent the problems related to the selection of specific appropriate transgenic lines (mostly due to integration position effects) and nonspecific activation of genes or pathways through nontarget interactions. This article reports the development of a method for functional testing of TFs using a robust *Agrobacterium*-based transformation protocol using embryonic spruce cells, which are easily cultured to facilitate the screening of a large number of genes directly in a conifer. This system enables the simultaneous introduction of TF-expression vectors and promoter–reporter gene constructs into spruce and evaluation of resulting gene expression on a weekly basis. Over 600 functional assays were carried out with 59 full-length cDNAs encoding TFs cloned into plant expression vectors and putative promoter sequences for 12 candidate genes from *Picea glauca*. The portfolio of TFs and promoters was developed from the *Picea glauca* gene catalogue (Rigault *et al.*, 2011). The current work reports the identification of several TF–promoter combinations showing *trans*-activation using this experimental system. Sequence analyses, expression clustering profiling and clustering, and *trans*-activation results were integrated to identify a putative spruce NAC TF proposed to control transcriptional activation for genes involved in SCW formation in *Picea glauca*.

## Materials and methods

### Plant material

For transcript profiling, 3-year-old white spruce seedlings obtained from open-pollinated seed lots were transferred to 2-l pots and grown in a greenhouse under natural photoperiod from mid-April to the end of June. The plants were watered every 2 d on average and were fertilized weekly with 20g l^–1^ N-P-K. Destructive sampling of 48 plants involved four time points: 6 am and 3 pm on 29 June (day 1) and 30 June (day 2), 2010. Nine vegetative tissue samples were collected from each tree: apex (AX, top 5mm, main stem with needles), secondary xylem (XS, main stem formed in the previous year), root xylem (XR, woody tissue of the largest roots), phelloderm (PS, bark and secondary phloem of the main stem formed in the previous year), root periderm (PR, from the largest roots), elongating shoot (ES, annual growth without needles), root tips (RT), young needles (YNS, foliage on annual growth of the main stem; YNB, from branches). At each time point, 16 plants were sampled and the tissues from four trees were pooled to form a biological replicate (*n* = 4). Samples were quickly frozen in liquid nitrogen and stored at –80 °C until further use.

### RNA extraction, cDNA preparation, and quantitative PCR analysis

Total RNA was isolated following the method of Chang *et al.* (1993) with modifications described previously (Pavy *et al.*, 2008). RNA concentration and integrity were determined using a NanoDrop 1000 (Thermo Scientific, Wilmington, DE, USA) and an Agilent 2100 Bioanalyzer (Agilent Technologies, Santa Clara, CA, USA), respectively. Complementary DNA (cDNA) was synthesized with the SuperScript First-Strand Synthesis System for RT-PCR (Invitrogen, Carlsbad, CA, USA), following the manufacturer’s instructions with minor modifications. Briefly, 1 μg total RNA was reverse transcribed using an anchored oligo d(T), and a GFP spike-in was added as an internal control of the reverse transcription. The cDNAs were diluted 1:4 in RNase-free nanopure water. Quantitative PCR was essentially as per Boyle *et al.* (2009) with some modifications. Briefly, PCR mixtures contained either QuantiFast SYBR Green PCR kit (Qiagen, Germantown, MD, USA) or LightCycler 480 SYBR Green I Master (Roche, Basel, Switzerland) and were composed of 1× master mix, 300nM of each gene-specific primer, and 5 μl diluted cDNA in a final volume of 15 μl. Gene-specific primers (Supplementary Table S1 available at *JXB* online) were designed with Primer3Plus software (http://www.bioinformatics.nl/cgi-bin/primer3plus/primer3plus.cgi), verified for self-complementarity with the Oligonucleotide Properties Calculator (http://www.basic.northwestern.edu/biotools/oligocalc.html), and for specificity against the *Picea glauca* gene catalogue (Rigault *et al.*, 2011). Reaction specificity was verified by the presence of a single amplification product based on melting curve analysis. The number of molecules (transcripts) were calculated for each sample using linear regression of efficiency (Rutledge and Stewart, 2008) adapted for Excel (Boyle *et al.*, 2009), normalized using a ratio based on the geometric mean of six *Picea glauca* reference genes and the GFP spike-in (HM151400.1) and transformed to a log_2_ scale for statistical analysis. Melting curve analysis was performed to ensure the amplification of a single product. Samples whose amplification of primer–dimer or nonspecific amplification was equivalent to 50% or more of the amplification of the gene were removed from the analysis.

The reference genes were: *elongation factor 1a* (*EF1-α*, BT102965), *cell division cycle 2* (*CDC2*, BT106071), *ribosomal protein L3A* (BT115036), *eukaryotic initiation factor 4E* (BT112014), *ubiquitin-conjugating enzyme* (BT109864), and *core histone H2A/H2B/H3/H4* (BT116867) (Supplementary Table S2). The stability and the variation of the reference genes were analysed with the geNorm 3.5 algorithm (Vandesompele *et al.*, 2002) before using them for normalization (Supplementary Table S7).

### Analysis of expression data

Normalized quantitative PCR data of the 102 different genes were obtained for day 1 and were analysed to compare the morning and afternoon samples (for each gene and tissue separately) by using Student’s t-test, with Bonferroni’s step-down correction for multiple testing (Holm, 1979). Further analyses only included genes with no significant difference between morning and afternoon samples (adjusted *P*-value < 0.05), which were combined (within a tissue) for statistical tests. The effect of the tissue type was tested by using an individual one-way analysis of variance for each gene; *P*-values were adjusted with Bonferroni’s correction. Duncan’s multiple range test was used to determine preferential expression among the nine tissues. The analyses were conducted in the R statistical environment release 2.12.1. In addition, 15 genes were tested for consistency between day 1 and day 2. Strong and statistically significant Pearson’s coefficients (mean 0.86) were obtained between days for the 13 genes that varied between tissues, and no interactions were observed between day and tissue effects, even though the day effect was significant for some genes (Supplementary Table S3). In contrast, the two genes that did not vary between tissues were found to vary significantly between days and gave low (nonsignificant) Pearson’s coefficients. This work analysed only samples from day 1 given that tissue differential expression was repeatable over this time period.

The genes with significant tissue effect were clustered by using complementary methods in the MultiExperiment Viewer software release 4.2 (http://www.tm4.org; Saeed *et al.*, 2003). Prior to clustering, the scale of the data was transformed from numbers of molecules to relative expression values: for each gene, the tissue with the most RNA molecules was set to 100, the threshold of detection was set to 0, and a relative value (between 0 and 100) was calculated for other tissues based on their ratio to the highest sample. Hierarchical clustering of genes and tissues was used to represent tissue-preferential transcript accumulation. The self-organizing tree algorithm (SOTA) was used to cluster genes and define expression profiles according to the following parameters: maximum cell diversity 0.01, Euclidean distance, and a maximum of 12 cycles. Each gene was statistically tested for fit against the 12 SOTA vectors by using Pavlidis template matching (PTM) analysis with default parameters and a *P*-value of 0.05.

### Isolation of 5′-genomic sequences and construction of vectors

The 5′-upstream sequence of each candidate target gene’s coding region was identified using the Universal GenomeWalker kit (Clontech, Mountain View, CA, USA) as previously described (Germain *et al.*, 2012). Amplified fragments were cloned using the TA cloning kit (Invitrogen) and electroporated into *Escherichia coli* XL1-Blue. Promoters were subsequently amplified from *Picea glauca* genomic DNA using gene-specific primer pairs designed from the fragment obtained by genome walking (Supplementary Table S2) and then cloned and sequenced. The putative promoter regions and 5′-UTR of each candidate gene were submitted to GenBank (Table 2). The digested fragments (*Xba*I–*Bam*HI) were inserted into a modified pMJM vector (Levée *et al.*, 2009) to create promoter::GUS fusions as previously described in Bedon *et al.*, (2009). These fusion fragments were inserted into a modified pCAMBIA2300 at the *Sse*8387I site where the hygromycin resistance gene is removed and replaced by the silencing inhibitor p19 protein-encoding gene (Voinnet *et al.*, 2003) to improve transgene expression efficiency. TF-expression vectors were obtained by PCR amplification using cDNA full-length coding sequences. PCR products were transferred to a pCAMBIA2300 expression vector using the Gateway system (Invitrogen), where they were flanked by the ubiquitin promoter and the 35S terminator. The reporter and expression vectors were transferred to *Agrobacterium tumefaciens* AGL1.

### Transient *trans*-activation transformation and quantification analysis

A *trans*-activation assays system based on *Agrobacterium* cotransformation using *Picea glauca* somatic embryogenic cells was developed using an approach similar to a previously described one for *Arabidopsis* (Berger *et al.*, 2007). This transient transformation assay is derived from the initial steps of the stable transformation procedure described for spruce (Klimaszewska *et al.*, 2004) with conditions optimized to maximize and maintain the level of transient expression observed up to 6 d. Briefly, a liquid culture of *Picea glauca* embryogenic cells (line PG653) was maintained and subcultured weekly. Following an overnight incubation, cells from the two *Agrobacterium* cultures (promoter–reporter with TF or empty expression vectors) were harvested by centrifugation, resuspended in embryogenic liquid culture media, and each added to 10ml of 1-d-old suspension culture to a final OD of 0.3. The cocultures were then placed in a shaker for 1h at 21 °C after which the cells were evenly vacuum filtered onto four separate 4.3-cm filter papers. Each filter was then placed in a separate Petri dish on two 18-cm filter papers wetted with 7ml liquid media supplemented with 100 μM acetosyringone. The Petri dishes were then sealed and kept in the dark at 21 °C. After 6 d of coculture, each filter paper was vacuum pulsed to remove excess liquid and a histochemical GUS assay was performed (Klimaszewska *et al.*, 2004). The level of *trans*-activation on each filter was visually evaluated compared to the control without TF. For each of the TF–promoter combinations, the observed reporter gene expression was rated based on the number of filters showing increased GUS blue staining. *Trans*-activations were rated as positive when visually at least three of the four filters showed clearly increased expression of GUS by histological staining (relative to the control), or likely positive when only two filters showed clearly increased GUS expression. In the absence of increased GUS expression on any of the four filters, or when only one filter was positive, the test was evaluated as showing no *trans*-activation. For those promoters alone, which showed a sufficiently high level of background GUS expression, promoter–TF interactions that caused a reduction in observed promoter activity were evaluated as causing a downregulation.

### Sequence comparison and phylogenetic analysis

Full-length nucleotide sequences for 27 NAC genes were obtained from this group’s *Picea glauca* gene catalogue (Rigault *et al.*, 2011). Class IIB NAC-domain subfamily was defined using *Arabidopsis* and poplar members (Ohtani *et al.*, 2011). The NAM/NAC domain from deduced amino acid sequences was used for multiple alignment using ClustalW with MEGA 4.0.2 software (Tamura *et al.*, 2007), as previously described (Bedon *et al.*, 2010). Two neighbour-joining trees were constructed using MEGA 4.0.2 software using the following parameters: Jones–Taylor–Thornton substitution model, gamma parameter of 1.0, and pairwise gap deletion. Bootstrap consensus trees were deduced from 1000 replicates.

## Results

Transcript profiling and expression cluster analysis of a large number of TFs and their potential target genes allowed this work to identify groups of coordinately expressed genes with respect to wood formation. A portfolio of gene constructs of full-length coding sequences for several classes of TFs was developed as well as the promoter sequences of selected genes involved in different physiological processes related to wood formation. TF/promoter interactions were identified using a transient transformation system developed in an embryogenic white spruce cell culture.

### Clustering of transcript profiling data

**Fig. 1.**
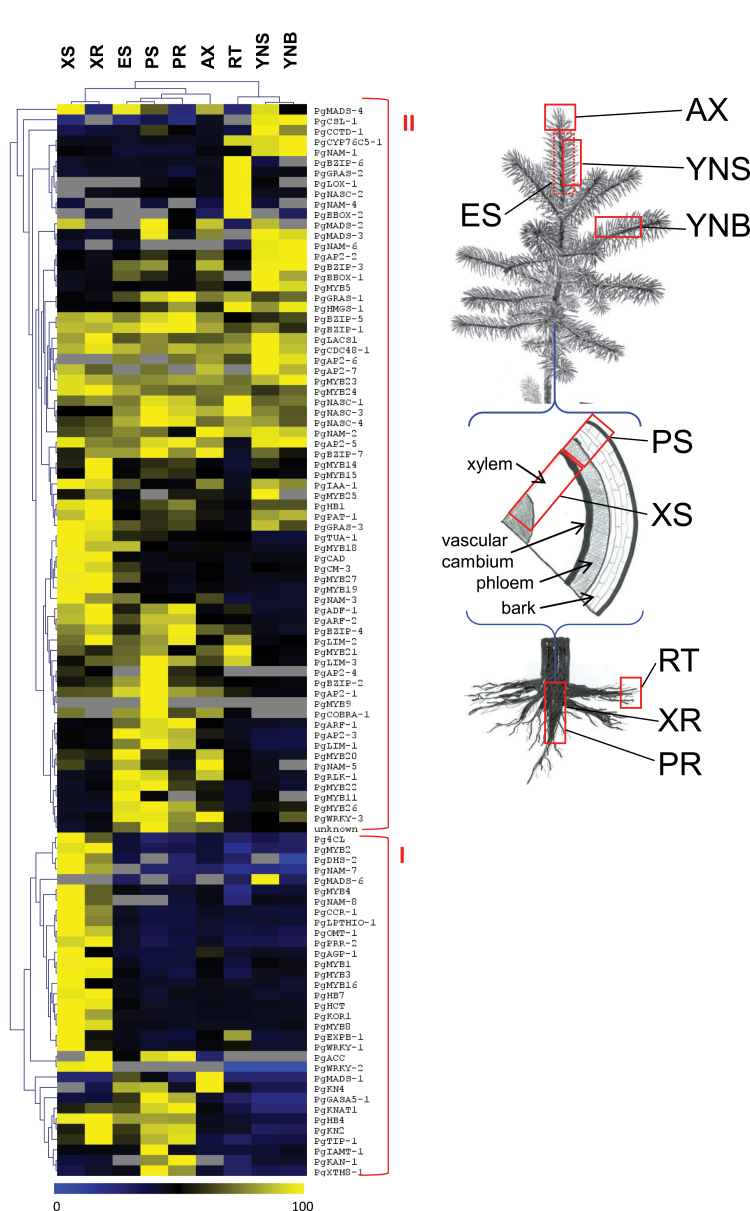
Hierarchical clustering showing tissue-preferential transcript accumulation. The clustering was based on relative transcript accumulation values (0–100) determined by quantitative PCR with gene-specific primers and based on a mean number of molecules normalized for each tissue. Genes that varied between sampling time points (morning and afternoon) are not shown (grey boxes). XS, secondary xylem from the main stem; XR, secondary xylem from largest roots; ES, elongating shoot (without needles); PS phelloderm (bark and secondary phloem) of the main stem; PR, periderm from largest roots; AX, shoot apex; RT, root tips; YNS, young needles from the main stem; YNB, young needles from branches.

High-quality transcript accumulation data were obtained for a set of 102 TFs and candidate genes in nine different vegetative tissues or organs from *Picea glauca* (Supplementary Table S6). A large majority of the genes tested (82/102, 79%) showed statistically significant variations between the nine different sample types (ANOVA *P*<0.05 with correction for multiple tests). A much smaller proportion (19%) varied significantly between the morning and afternoon in one or more sample types (Student’s t-test, *P*<0.05 with correction). There was little overlap between the tissue preferential genes and the genes that varied between the morning and afternoon samples. Hierarchical clustering was applied to the 82 genes that varied between tissue and organ samples to visualize putative groups of coexpressed genes. The genes were clustered into two major expression groups (Fig. 1, left panel): one cluster contained genes with a clear preferential accumulation either in the secondary xylem of the stems and roots or in the phelloderm and periderm (Fig. 1, left panel, bottom). A second and larger cluster of genes included a variety of profiles, with moderately preferential expression in various tissue types (Fig. 1, left panel, top).

The expression data were analysed with SOTA followed by PTM to define statistically robust coexpression groups for the 82 tissue preferential genes. The SOTA analysis grouped the genes into five major expression clusters comprising between six and 25 members (clusters 1, 2, 3, 7, and 9; Supplementary Fig. S1), and seven minor clusters containing between one and three members. The PTM analysis showed similarity among the SOTA clusters: 69 genes fitted at least one and up to five profiles with statistical confidence (*P*<0.05). Most notably, 27 genes matched three or four of the clusters 9, 10, 11, and 12 (although some also matched up to two other clusters). The TFs in this set of transcripts included eight of the R2R3-MYBs genes (*PgMYB1*, *PgMYB4*, *PgMYB8*, *PgMYB2*, *PgMYB3 PgMYB16*, *PgMYB19*, and *PgMYB27*), two HD-ZIP III (*PgHB7* and *PgHB8*), and three NAC (*PgNAC-3*, *PgNAC-7*, highly preferential to xylem, and *PgNAC-8*) that were preferential to secondary xylem tissues from stems and roots. They were coexpressed with candidate genes encoding lignin biosynthesis enzymes (Pg4CL, PgCAD, PgCCR-1, PgHCT, PgOMT-1) and cell-wall proteins (PgKOR1, PgAGP-1).

### Development of a portfolio of conifer TFs and specific candidate gene promoters

Based on predicted gene functions and expression data clustering, a portfolio of spruce TF-expression vectors and of promoter::GUS constructs for candidate genes was developed for screening in functional assays. This work focussed on 13 TF families (Table 1) from the collection of full-length *Picea glauca* cDNAs in which 927 unique transcript sequences have been assigned to 34 plant TF families (Rigault *et al.*, 2011). The complete list and annotations are presented in Supplementary Tables S4 and S5. Members of the selected families have been linked to development, including vascular growth and differentiation such as NAC, MADS, AUX\IAA, to stress responses such as AP2, WRKY, or to both of these biological processes, such as b-Zip and MYBs. Transcript profiling and activity screening experiments covered an overlapping set of 59 TF cDNA clones, each containing a complete coding sequence as determined by large-scale cDNA sequencing (Rigault *et al.* 2011) and targeted TF analyses in conifers (Bedon *et al.*, 2007, 2010; Bomal *et al.*, 2008).

**Table 1.** Selected TF families used for *trans*-activation assaysAP2: APETALA2 domain; Aux/IAA: auxin/indole-3-acetic acid proteins; bZIP: basic region/leucine-zipper motif; GRAS: GIBBERELLIN ACID INSENSITIVE (GAI), REPRESSOR of GA1 (RGA), and SCARECROW (SCR); Homeobox: homeodomain; KNOX2: KNOX2 domain proteins; LIM: LIM domain; MADS: MCM1, Agamous, Deficiens, Srf; MYB: Myb-like DNA-binding domain; NAC: NAM, ATAF1/2, and CUC2 domain; WRKY: WRKY DNA-binding domain.

Family	cDNA identified	Full-length coding sequence	Tested interactions
AP2	63	21	93
AUX/IAA	26	7	24
B-box zinc finger	6	6	8
bZIP	31	11	64
GRAS	17	4	20
Homeobox	54	9	8
KNOX2	7	3	24
LIM	5	4	32
MADS	45	18	70
MYB*	122	42	201
NAC domain	36	19	84
NASC	7	6	44
WRKY	25	12	36
Total	444	162	708

The choice of candidate genes for promoter isolation was based on functional data from previous experiments, expression data, and scientific literature indicating they may be downstream targets for the TFs under study. Overall, this study selected genes that have been linked to vascular growth and differentiation in trees, including those encoding for lignin biosynthesis enzymes (Pg4CL, PgCAD), proteins involved in cell-wall synthesis and remodelling (PgCesA-3, PgXTH8-1, PgTUA-1, alpha-tubulin-1), and transcriptional regulators (PgHB4, PgLIM-1, PgMYB1, PgMYB8; Table 2) based on several previous reports (Plomion *et al.*, 2001; Peter and Neale, 2004; Bomal *et al.*, 2008; Paiva *et al.*, 2008; Bedon *et al.*, 2010). The list also included wound-inducible and defence-response genes (*PgCAD, PgDHS2*; Bedon *et al.*, 2009). The putative promoters of these 12 genes were identified as the region immediately upstream (5′) of their coding sequences (Table 2). The lengths of the isolated promoters were from 931 to 3620bp.

**Table 2.** Candidate genes used for promoter isolationSize includes the promoter region and the 5′-UTR.

Candidate gene	Description	Function	Size (*n*)	GenBank accession no.	References
PgDHS2	3-Deoxy-7- phosphoheptulonate synthase	Enzyme of the shikimate pathway; provides precursors for monolignol and flavonoid metabolisms	1353	JN828804	Bomal *et al.* (2014)
Pg4CL	4-Coumarate CoA ligase	Lignin synthesis	1885	JN828803	Bomal *et al.* (2014)
PgCAD	Cinnamyl alcohol dehydrogenase	Lignin synthesis	1519	FJ428229	Bedon *et al.* (2009)
PgCesA-3	Cellulose synthase A	Cellulose synthesis in secondary cell walls	2085	KF824520	Taylor (2008) ^*b*^
PgHB4	Class III homeodomain leucine zipper (HD-Zip III	Transcriptional regulation; involvement in primary and secondary vascular tissue pattern formation	2407	KF834195	Côté *et al.* (2010)
PgLIM-1	LIM (LIN11/ISL-1/MEC-3) TF family	Transcriptional regulation of genes related to secondary cell walls	1780	KF834197	Kawaoka and Ebinuma (2001) ^*b*^; Demura and Fukuda (2007) ^*b*^
PgMYB1	MYB domain TF	Transcriptional regulation of genes related to secondary cell walls	3620	KF834198	Bedon *et al.* (2007); Bomal *et al.* (2008)
PgMYB8	MYB domain TF	Transcriptional regulation of genes related to secondary cell walls	2332^*a*^	KF834199	Bedon *et al.* (2007); Bomal *et al.* (2008)
PgGASA5-1	Gibberellic acid-stimulated *Arabidopsis*	Gibberellin-responsive protein; putative role in cell expansion in wood-forming tissue of *Pinus pinaster*	995	KF834196	Paiva *et al.* (2008) ^*b*^
PgSABATH2	SABATH family methyl transferase	Putative role in phytohormone methylation	2179	KF834200	Zhao *et al.* (2009)
PgTUA-1	α-Tubulin	Cytoskeleton and cell-wall organization	944	KF834201	Lloyd and Chan (2008) ^*b*^
PgXTH8-1	Xyloglucan endotransglycosylase/ hydrolase	Modification of the xyloglucan-cellulose framework of plant cell walls; control of cell-wall expansion and strength	931	KF834202	Mellerowicz *et al.* (2008)^*b*^

^*a*^Includes one intron of 278bp.

^*b*^General references about putative gene function.

### Functional screening for TF–promoter *trans*-activation

**Fig. 2.**
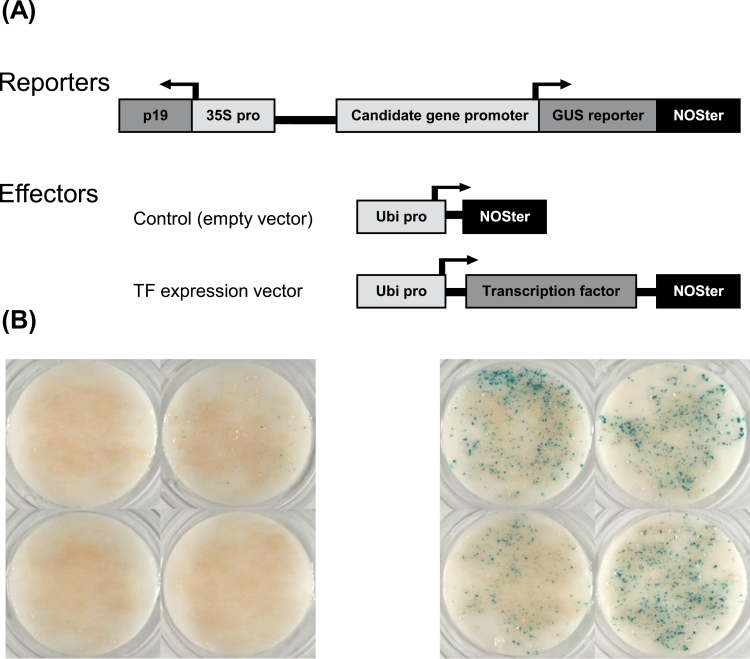
Overview of the components of the *trans*-activation method. (A) Schematic diagrams of the effector and reporter plasmids. The reporter constructs consisted of a GUS reporter gene driven by the tested candidate gene promoter; *p19* driven by the CaMV 35S promoter was inserted into the same plasmid. The effector constructs consisted of the Gateway-inserted complete coding sequence of each TF driven by the maize ubiquitin promoter. The control (empty vector) consisted of the expression vector without TF insertion. (B) Representative positive *trans*-activation. Histochemical GUS assay was performed after 6 d of embryogenic cells/agrobacteria coculture. Left: control assay of white spruce cells transformed with the *PgMYB8* promoter-reporter construct and empty vector effector construct; right: white spruce cells transformed with the *PgMYB8* promoter-reporter construct and the *PgNAM-7* effector construct.

An *Agrobacterium* transient transformation method was developed for spruce and was used to test 664 combinations comprising a TF and a candidate target gene promoter. As illustrated in Fig. 2, the method used *Picea glauca* embryogenic cell cultures that were cotransformed with *Agrobacterium*: one culture containing a promoter::GUS construct and the other culture either with a full-length coding sequence of a selected TF (to test for interaction) or an empty vector (control).

**Fig. 3.**
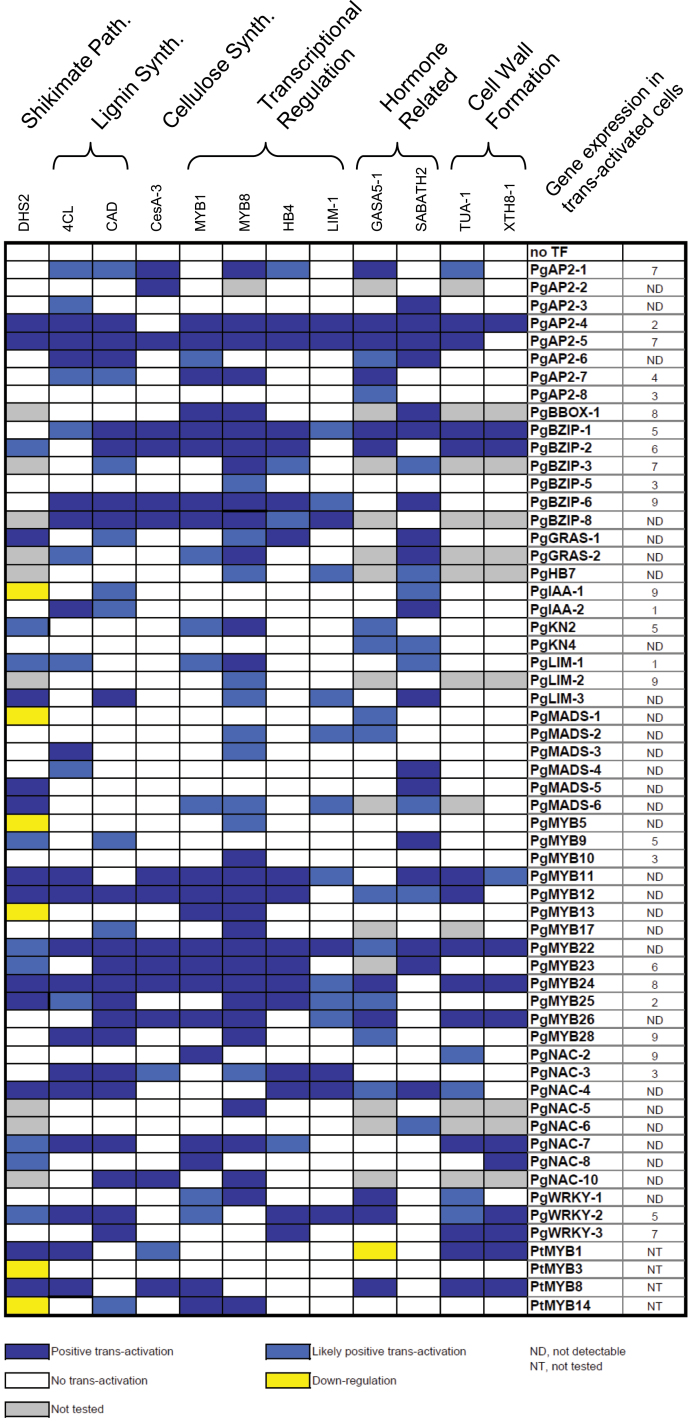
Transient *trans*-activation assays leading to the identification of specific interactions. Each box represents the observation of the histochemical test compared with the control without TFs. The *PgDHS2* and *PgGASA5-1* promoters alone showed faint GUS staining and loss of expression (relative to the control) was observed with specific TFs (yellow). Relative gene expression levels for each TF based on PiceaGenExpress are indicated on the right. This database was developed from transcript profiles obtained for eight different tissue types coming from five independent experiments (Raherison *et al.*, 2012). Briefly, the genes were ranked based on their signal intensities within a tissue type and equally divided into 10 separate classes according to their signal intensity: class 1: 10% lowest signal intensities; class 10: 10% highest signal intensities (see Raherison *et al.,* 2012 for details). None of the genes tested were ranked in class 10.

Fig. 3 presents an assessment of GUS staining following each of the 664 cotransformation experiments, with each interaction tested in quadruplicate. Positive combinations are those in which the addition of the TF modified the level of GUS staining with a given promoter::GUS construct. In total, this study observed 192 clear positive combinations, representing 29% of all the combinations tested. Promoter–TF combinations with positive GUS staining in two of the replicates suggested weak or conditional interactions and represented 13% of the tested interactions. No *trans*-activation was observed when promoters were cotransformed with empty vectors (no TF), except for *PgDHS2* and *PgGASA5-1* promoters, which showed significant and consistent background GUS expression. A clear repressive effect (1% of interactions) by a few TFs was observed with the *PgDHS2* and *PgGASA5-1* promoters (Fig. 3).

Gene expression levels of the TFs in spruce embryogenic cells were obtained from the PiceaGeneExpress database (Raherison *et al.*, 2012) and indicated that most of the TFs were among the 15% genes not detected by microarray analysis and only seven genes of the 33 tested were shown to be strongly expressed (Fig. 3, expression class >7). These observations indicated that the potential contribution of endogenous TF expression to *trans*-activation was small overall considering that recombinant TFs are expected to be expressed at much higher levels than endogenous factors in transiently transformed cells. Overall, the data strongly suggest that the method enabled the identification of TFs that acted on the candidate gene promoters.

### Identification in conifers of a putative transcriptional regulation network similar to the SND1/VND cascade

The development of a rapid method for screening TF–promoter interactions in a gymnosperm allowed us to survey the potential role of several members of the NAC-domain TF family in vascular growth in conifers. Functional studies of *Arabidopsis* NAC-domain proteins such as NST members (NAC SCW-thickening promoting factors) and SND members (SCW-associated NAC-Domain) have indicated that they are key transcriptional switches governing SCW biosynthesis and formation of vascular vessels, respectively (Demura and Fukuda, 2007; Yamaguchi *et al.*, 2011; Wang and Dixon, 2012). The current study investigated eight genes from the NAC-domain family to assess whether specific members with a predominant role in SCW formation may be found in gymnosperms. *PgNAC-4* and *PgNAC-7* gave positive interactions with the promoters of genes encoding enzymes of biosynthetic pathways related to SCW deposition (*PgCAD*, *Pg4CL*, *PgDHS2*, and *PgXTH8-1*) and genes encoding TFs expressed in wood-forming tissue (*PgLIM-1* and *PgHB4* for *PgNAC-4*; *PgMYB1* and *PgMYB8* for *PgNAC-7*). This observation is significant because transgenic *Picea glauca* plants overexpressing their pine orthologues, *PtMYB1* and *PtMYB8*, had enhanced lignification and developed ectopic SCWs, to a greater extent in PtMYB8 lines (Bomal *et al.*, 2008). The pine genes *PtMYB1* and *PtMYB8* were also tested in this transient system and were able to positively regulate promoters from genes directly related to SCW synthesis such as *PgCesA-3*, *Pg4CL*, and *PgXTH8-1* (Fig. 3). *PtMYB8* was able to positively regulate the *PgMYB1* promoter, suggesting that *PgMYB1* may be downstream of *PgMYB8*. These results suggest that PgNAC-7 may be a TF that acts as an upstream regulator targeting *PgMYB1* and *PgMYB8*, which in turn positively regulate genes encoding enzymes of SCW formation.

### Identification of two conifer NAC-domain TFs similar to their orthologous proteins from angiosperms

**Fig. 4.**
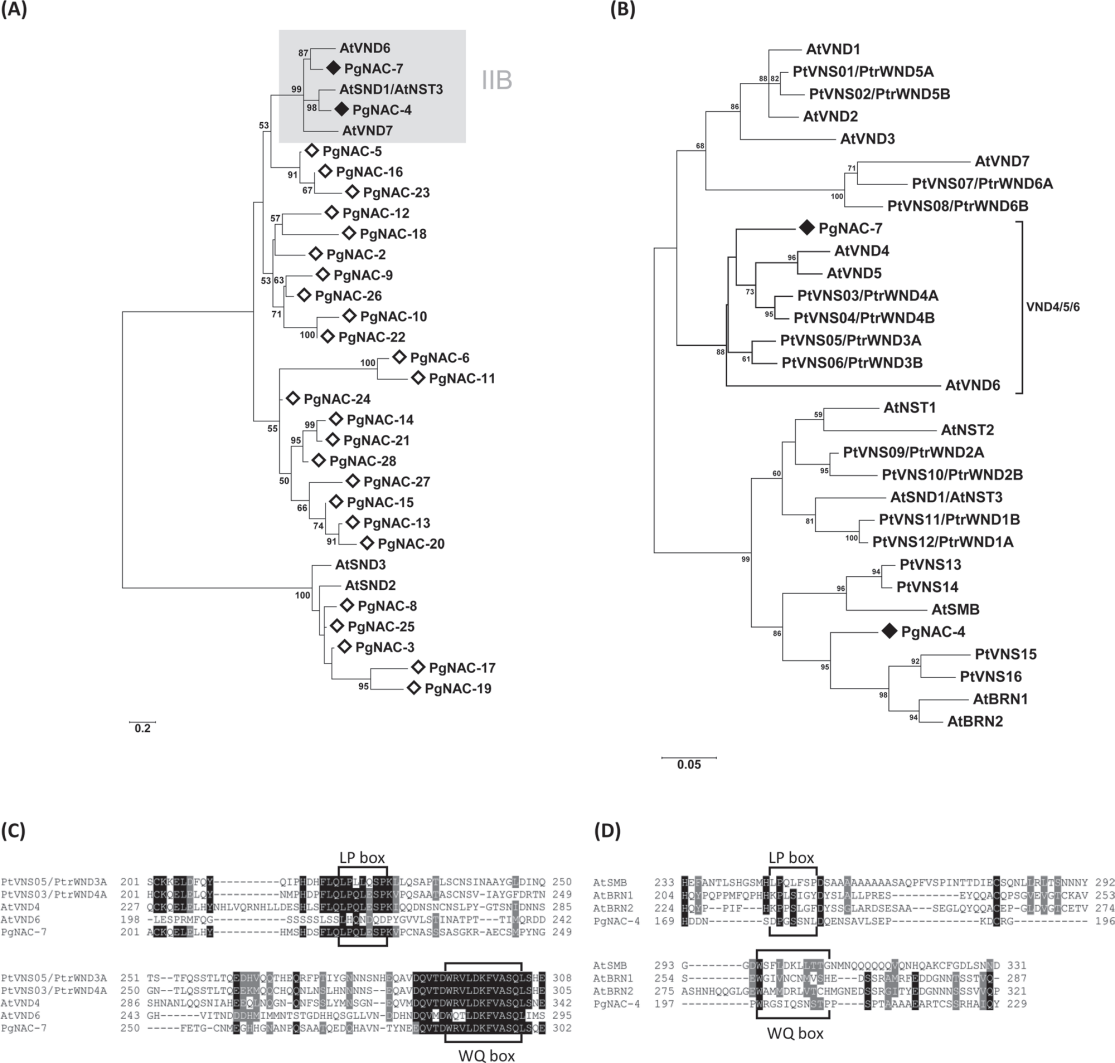
Identification of conifer NAC-domain proteins similar to the vascular proteins from angiosperms. (A) Unrooted neighbour-joining phylogenetic tree of NAC-domain proteins identified in *Picea glauca*. *Arabidopsis* proteins were chosen as landmarks representing the vascular NAC-domain IIB subfamily (grey box). All *Picea glauca* NAC-domain proteins are indicated by open diamonds and those identified in the IIB subfamily are indicated by filled diamonds. (B) Unrooted neighbour-joining phylogenetic tree of NAC-domain proteins from *Arabidopsis*, poplar, and *Picea glauca* identified in the IIB subfamily. *Picea glauca* proteins are indicated by filled diamonds. (C) Multiple sequence alignment of the C-terminus domains between PgNAM-7 and members of the VND subfamily; LP and WQ motifs are indicated. (D) Multiple sequence alignment of the C-terminus domains between PgNAM-4 and members of the SMB/BRN subfamily; LP- and WQ-like motifs are indicated. At: *A. thaliana*; Pg: *Picea glauca*; Pt and Ptr: *Populus trichocarpa* sequences, published by Ohtani *et al.* (2011) and Zhong *et al.* (2010b), respectively.

Previous studies reported that NAC-domain TFs involved in vascular development form the class IIB clade that includes NST1/2/3, VND1-7, SMB, and BRN1/2 (Kubo *et al.*, 2005; Mitsuda *et al.*, 2005; Zhong *et al.*, 2006; Bennett *et al.*, 2010). Here, a multiple alignment of NAC-domain sequences (N-terminus DNA-binding domain) and construction of a phylogenetic tree showed that PgNAC-4 and PgNAC-7 are the only *Picea glauca* NAC-domain TFs (within the 31 identified) that belong to the class IIB clade (Fig. 4A). PgNAC-4 was part of the BRN/SMB subgroup whose proteins were shown to play a role in root cap differentiation (Bennett *et al.*, 2010). In contrast, PgNAC-7 showed higher homology with the VND family, particularly with VND4 and VND5 (Fig. 4B). Members of the IIB NAC-domain family contain two characteristic motifs within their transcriptional activation domain located at the C terminus (i.e. the LP and WQ boxes necessary for their activity; Ko *et al.*, 2007). The protein sequence of the PgNAC-7 C termini contained canonical LP and WQ motifs (Fig. 4C) identical to those in AtVND4 and PtVNS03/PtrWND4A. PgNAC-4 contained sequences related to LP and WQ motifs, mostly represented by the canonical proline and tryptophan as in the *Arabidopsis* genes *AtSMB*, *AtBRN1*, and *AtBRN2* (Fig. 4D).

**Fig. 5.**
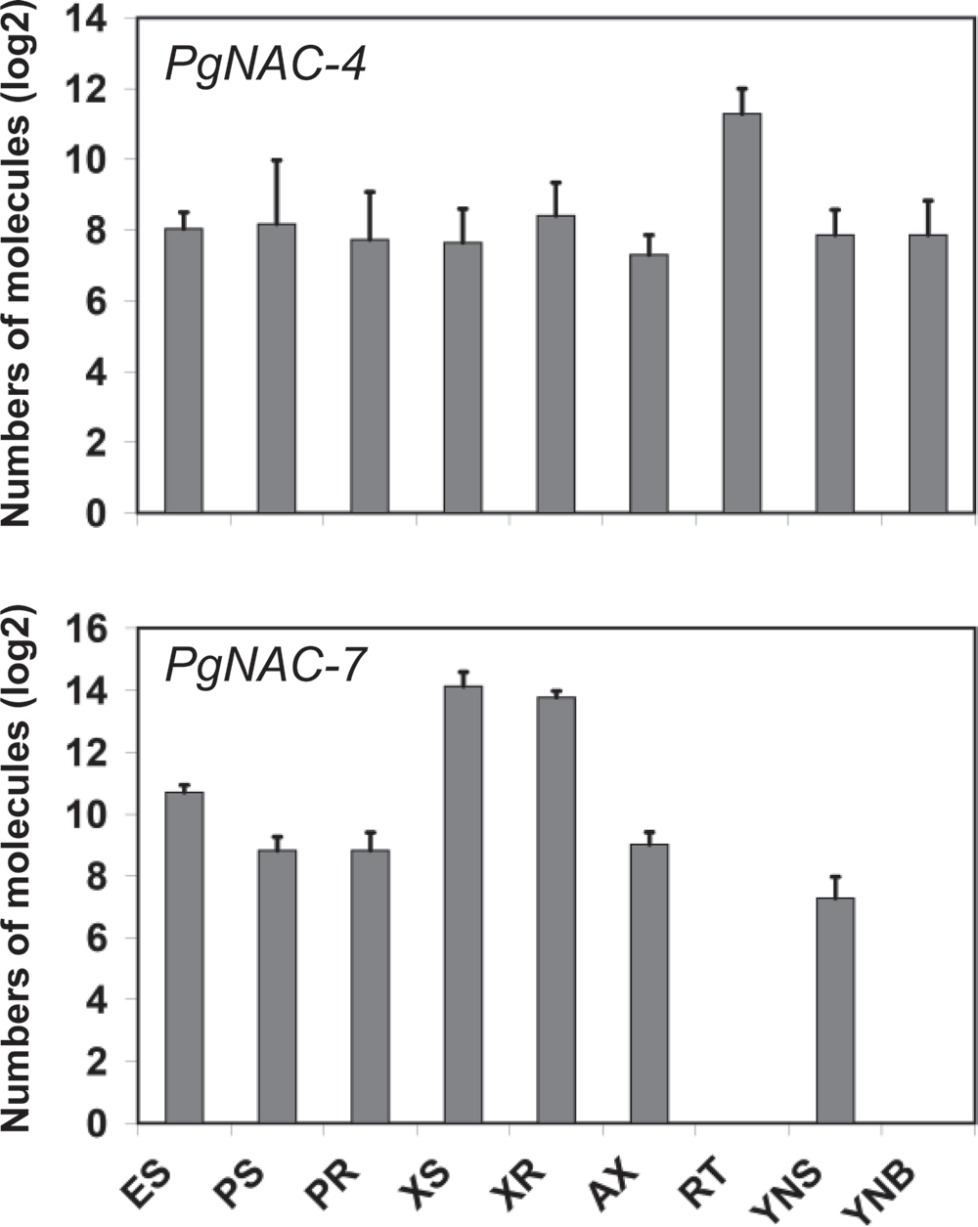
Transcript profiles of PgNAC-4 (top) and PgNAC7 (bottom) in white spruce. Transcript levels were determined by quantitative PCR with gene-specific primers. Data are mean standard deviation from three or four biological replicates, each comprised by pooling tissues from four trees, and are presented as log_2_ number of RNA transcript molecules per ng of total RNA; differences of 2, 3, 4, or 5, represent 4-, 8-, 16-, or 32-fold differences, respectively. ES, elongating shoot (without needles); PS, phelloderm (bark and secondary phloem) of the main stem; PR, periderm from largest roots; XS, secondary xylem from the main stem; XR, secondary xylem from largest roots; AX, shoot apex; RT, root tips; YNS, young needles from the main stem; YNB, young needles from branches.

Expression profiling showed 8–120-fold higher transcript accumulation for *PgNAC-7* in secondary xylem of both roots and stems compared to the other vegetative tissues and organs tested (Fig. 5). This finding is consistent with observations reported for the VND4/5/6 subfamily in *Arabidopsis* (Kubo *et al.*, 2005). Similarly, *PgNAC-4* transcript levels were relatively uniform in most tissues but were 8–10-fold higher in the root tip (Fig. 5), also consistent with *AtBRN1* and *AtBRN2* expression patterns in *Arabidopsis* (Bennett *et al.*, 2010). Analysis of transcript accumulation for spruce TFs and candidate genes in the present study indicated that *PgNAC-7* shared tissue profiles also found for genes directly involved in secondary growth.

## Discussion

### Coordinated expression of TFs and candidate genes in specific *trans*-activation patterns

Despite the rapid development of genomic tools for conifers such as genetic maps, expressed sequence tag databases, and DNA microarray systems, functional characterization of candidate genes is challenging. Screening for mutants is an extremely tedious task and the long breeding time makes forward genetic approaches impossible. *Populus* has been a model tree species for studies of gene function because of ease of *in vitro* cultivation and genetic transformation, including activation tagging (Harrison *et al.*, 2007). For conifers such as pines and spruces, efficient genetic transformation procedures have been developed (Tang *et al.*, 2001; Klimaszewska *et al.*, 2004). Nevertheless, many conifer genes have been studied in model plants such as *Arabidopsis* and tobacco because of the long regeneration times and the technical complexity of producing transgenic conifers (Patzlaff *et al.*, 2003b; Newman *et al.*, 2004).

Only a limited number of genes have been functionally analysed by using a homologous expression system in coniferous species. Some involve misexpression analyses of a gene encoding an enzyme to reveal its potential function in a metabolic pathway such as lignin (Wadenbäck *et al.*, 2008; Wagner *et al.*, 2009) and terpene biosynthesis (Hamberger *et al.*, 2011). Molecular mechanisms underlying somatic embryo development in spruce have also been investigated (Belmonte *et al.*, 2007; Klimaszewska *et al.*, 2010). In the current group’s laboratory, functional characterization has been initiated for TFs belonging to a few different classes and has linked two MYBs to SCW lignification and related gene deregulation (Bomal *et al.*, 2008; Bedon *et al.*, 2010). Spruce overexpression lines for both *PtMYB1* and *PtMYB8* displayed ectopic lignification and delayed growth and upregulation of genes encoding enzymes from the lignin biosynthesis pathway (Bomal *et al.*, 2008).

Depending on research objectives, transient transformation can be a valuable alternative offering several advantages compared with stable transformation. Although it cannot reveal morphological phenotypes such as cell-wall composition or vascular tissue architecture, it represents a rapid approach to obtaining indications of TF–promoter interactions. To date, *Arabidopsis* and tobacco protoplast transfections have been extensively used as transient expression systems. Nevertheless, protoplast transfection systems have been developed for emerging model systems such as *Physcomitrella patents* (Thévenin *et al.*, 2012) that are fast, versatile, and robust allowing elucidation of specific TF roles in signalling activities and cellular processes. The current work aimed to develop a homologous transient transformation method in spruce for qualitative testing of promoter–TF *trans*-activations on a large scale. To achieve this goal, first a resource for testing potential interactions of TFs with several different promoters in conifers was developed by assembling a portfolio of spruce promoters and a large collection of the complete coding sequences of members of several families of spruce TFs. This method took advantage of the relative uniformity of the cells within embryogenic cell cultures and their compatibility with genetic transformation by *in vitro* cultivation with *Agrobacterium*. The *trans*-activation system developed here offers a distinct advantage to functional testing using a heterologous species (such as *Arabidopsis*) in that key cofactors required for TF function are more likely to be present in a homologous species. This, however, does not rule out the possibility that key factors unique to a specific tissue type such as developing secondary xylem may not be present in somatic embryonal cells. Nevertheless, it stands to reason that a homologous system would better reflect reality compared to the use of a yeast one-hybrid assay for instance.

This approach revealed positive interactions of promoters resulting from TF *trans*-activation. Most of the promoters that were tested did not show background expression that could result from endogenous expression of TFs. Some TF families have been shown to form complexes with other proteins in order to activate properly their target genes. This was found to be particularly important in the regulation of specific genes involved in the anthocyanin biosynthetic pathway where a number of MYB TFs were shown to form ternary complexes with basic helix–loop–helix and WDR type proteins (Hichri *et al.,* 2011). It is possible that the lack of promoter–TF *trans*-activation is due to the absence of required cofactors. However, the method described here could easily accommodate for the introduction of a third gene construct encoding a specific cofactor, in addition to the TF-coding region and the promoter–reporter constructs. Also, any artificial *trans*-activation system is prone to false positives and this includes the one described here; the level of transcript accumulation of the tested TF is likely to be higher in the transiently transformed cells since strong promoters are commonly used. Also, this work cannot exclude the possibility that indirect or nonspecific promoter induction could result from high accumulation of the TF in the cell. However, this study group is quite confident about the robustness of this method as the data obtained confirms existing knowledge in the literature. As far as is known, the scope of the study greatly extends the number of TFs and promoter interactions that have been studied in any vascular plant species outside of flowering plants.

### Conifer class-IIB NAC-domain proteins display general features similar to *Arabidopsis*

In *Arabidopsis*, NAC-domain proteins (SND1, NSTs, and VNDs) orchestrate a transcriptional cascade that involves R2R3-MYBs and regulates vascular differentiation and SCW formation. Functional orthologues of members of this network described have been identified in poplar and eucalyptus (Zhong and Ye, 2009; Zhong *et al.*, 2010a). For example, poplar and eucalyptus NAC-domain proteins similar to AtNSTs/AtVNDs have been characterized through their expression patterns and restoration of WT phenotype in *Arabidopsis* mutants. Moreover, WNDs and MYB proteins in poplar and eucalyptus were shown to be directly involved in the transcriptional cascade described in *Arabidopsis*, suggesting a relative conservation of a core regulatory network related to SCW biosynthesis (Zhong and Ye, 2009; Zhong *et al.*, 2011). It was suggested that a similar cascade may also regulate SCW formation in conifers (Bomal *et al.*, 2008), but until the current report, functional evidence had only been obtained for MYBs (MYB1 and MYB8 from pine and spruce).

This report shows that *PgNAC-4* and *PgNAC-7* are the only two NAC-domain genes that clustered within the IIB subfamily. As such, they represent candidate NAC genes that could participate in a cascade regulating vascular development analogous to that described in angiosperm plants and trees. The data show that the PgNAC-7 amino acid sequence uniquely clusters with AtVND 4/5/6 and with poplar orthologues PtrVDN 3(A,B) and 4(A,B) and is distinct from other VNDs from *Arabidopsis* and poplar (Fig. 4). Its expression is strongly preferential to secondary tissue in both stems and roots (Fig. 5) and its transient expression activates the expression from promoters of MYBs (*MYB1* and *MYB8*) that have been linked to SCW formation, as well as downstream proteins and enzymes involved in cell-wall assembly (PgCAD, Pg4CL, PgTUA-1, PgXTH8-1; Fig. 3).

In contrast, PgNAC-4 specifically clustered with the SMB/BRN subclass genes AtSMB, AtBRN1, and AtBRN2 involved in cell-type specification and SCW modification during root cap maturation in *Arabidopsis* (Bennett *et al.*, 2010). *PgNAC-4* transcripts were found in all of the *Picea glauca* tissues tested but were clearly preferential (8–10-fold difference) to root tips (Fig. 5). In the *trans*-activation assays, PgNAC-4 gave positive interactions with promoters from genes related to SCW formation (*PgCAD*, *Pg4CL*, *PgDHS2*) as did PgNAC-7. However, the TFs activated by PgNAC-4 are different from PgNAC-7. PgNAC-4 was able to positively regulate *PgHB4* and *PgLIM-1* promoters but no significant inductions were observed for the *PgMYB1* and *PgMYB8* promoters (as observed for PgNAC-7). Tissue profiling also showed that *PgNAC-4*, *PgCAD*, *Pg4CL*, *PgDHS2*, *PgHB4*, and *PgLIM-1* do not share similar tissue specificity (Fig. 1). As only *PgNAC-4* transcripts were found in root tips (not *PgNAC-7*), it may be that regulation of *PgCAD*, *Pg4CL*, *PgDHS2*, *PgHB4*, and *PgLIM-1* by PgNAC-4 is restricted to a small part of the plant. Furthermore, conifer NAC-domain TFs from the IIB family may share the same generic ability to activate genes related to the SCW as observed in *Arabidopsis* for AtSMB, AtBRN1, and AtBRN2 (Bennett *et al.*, 2010).

### White spruce vascular NAC-domain TF master switches

This work identified two of the eight putative NAC-domain proteins as members of the IIB subfamily, whose members are known to regulate SCW biosynthesis. Rigault *et al.* (2011) showed that the family of NAC-domain proteins is one of the most underrepresented in *Picea glauca*, with 36 putative members compared with 113 and 162 in *Arabidopsis* and poplar, respectively. This observation is also supported by Lang *et al.* (2010) who showed that TF-encoding genes have diversified concomitantly with morphological complexity for the development of flowering plants. In angiosperms, water transport is carried out primarily by tracheary elements, and mechanical support is provided by vascular and intervascular fibre cells. In contrast, both of these functions are carried out by tracheids in gymnosperms (Raven *et al.*, 2005). Studies of SCW regulation in angiosperms showed that they have transcriptional regulators that may be specific to particular cells, such as NSTs in fibres and VNDs in vessels (Zhong *et al.*, 2010a). It was suggested that this specificity may be the result of the diversification and specialization of vascular tissues in angiosperms. Strikingly, the only NAC-domain protein identified to date in *Picea glauca* that could play a master regulatory role (PgNAC-7) is clustered in the VND-like subclade, which is specific to vessels. Furthermore, no NST-like gene sequences have been identified to date.

**Fig. 6.**
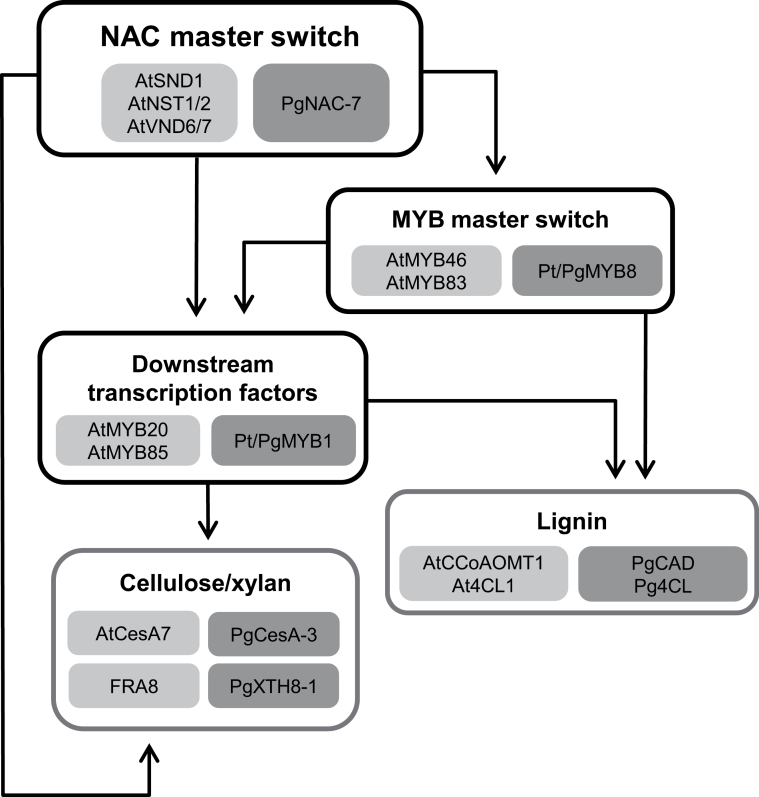
Transcriptional regulatory network controlling secondary cell wall (SCW) biosynthesis. Light grey boxes indicate *Arabidopsis* major components of the coordinated network leading to synthesis of the three major SCW constituents. Dark grey boxes indicate *Picea glauca* putative orthologues (transcription factor putative orthologues and genes encoding enzymes depicting the three major SCW biosynthesis pathways) and the putative transcriptional network identified in the present study.

The data presented in this report are helping to delineate putative transcriptional networks for the regulation of vascular tissue differentiation in *Picea glauca* based on sequence, expression, and functional similarity with angiosperm genes (Fig. 6). It was previously observed that PtMYB8 and, to a lesser extent, PtMYB1 may positively regulate genes involved in lignification such as *Pg4CL* and *PgDHS2* (Bomal *et al.*, 2008). PtMYB8 and PgMYB8 are close orthologues of AtMYB46 (Bedon *et al.*, 2007), which is a key regulator in xylem differentiation that can be directly regulated by AtSND1/NST3, NST1, NST2, VND6, and VND7 (Zhong *et al.*, 2007). More specifically, AtMYB46 has been identified as a MYB master switch for secondary growth. MYB1 from both spruce and pine are highly similar to AtMYB85 and AtMYB20 (Bedon *et al.*, 2007) and can be *trans*-activated by PgNAC-7. Moreover MYB20 and MYB85 in *Arabidopsis* have been shown to be positively regulated by AtSND1/AtNST1, and AtMYB85 is able to induce the *At4CL1* promoter (Zhong *et al.*, 2008).

An orthologous cascade of *trans*-activations has been recently identified in poplar with the characterization of WNDs acting as transcriptional master switches (McCarthy *et al.*, 2010; Zhong *et al.*, 2010b, 2011; Ohtani *et al.*, 2011). Despite the fact that some downstream activated TFs were identified as being involved in wood formation, about 13 new TFs (not related to secondary growth) have been identified in transgenic poplar overexpressing WND genes. They include *PtrWRKY12* and *PtrWRKY13* that are specifically expressed in vascular tissue (Zhong *et al.*, 2011). The current work showed that *PgWRKY-1* is coregulated with *PgNAC-7* and is highly preferential to secondary xylem (Fig. 1). Interestingly, the *trans*-activation assays showed that PgWRKY-1, whose closest poplar homologues are PtrWRKY12 and PtrWRKY13, positively regulate *PgMYB8* and most likely *PgMYB1* (Fig. 3). However, *PgWRKY-1 trans*-activation by PgNAC-7 and the putative role of this conifer WRKY gene in secondary growth remain to be demonstrated.

Although relationships of orthology between conifers and *Arabidopsis* must be viewed as tentative given that the two lineages have been separated for around 300 million years, these results suggest that PgNAC-7 acts as a first level of transcriptional control in SCW biosynthesis. Seeing that PtMYB8 and PgMYB8 can positively regulate PgMYB1, they are proposed to function at a second level, upstream of other MYBs in a manner analogous to AtMYB46 (Zhong and Ye, 2009; Zhao and Dixon, 2011), as depicted in this work’s model of the transcriptional network regulating SCW biosynthesis and lignification (Fig. 6). PgMYB1 is functionally related to AtMYB85 with a predominant role in SCW formation. Recently, a negative-feedback loop has been shown for the regulation of *AtSND1* promoter activity. Three MYB proteins from the Sg4 clade were shown to be downstream targets of AtSDN1 and were also able to directly repress its promoter through binding to a specific *cis*-motif, thus creating a negative-feedback loop (Wang *et al.*, 2011). The current coexpression study showed that *PgMYB3* and *PgMYB16* are coregulated with genes identified in the putative regulatory cascade leading to SCW biosynthesis (Fig. 1). Interestingly, these two genes also belong to the Sg4 clade of MYB proteins (Bedon *et al.*, 2010). Moreover, the current work showed that PtMYB3, the pine orthologue for PgMYB3, is able to repress *PgDHS2* promoter in the *trans*-activation assay, indicating its potential repression activity (Fig. 3).

In summary, PgNAC-7 is cast as a strong candidate among the *Picea glauca* NAC-domain TFs for the role of master switch in regulating secondary vascular growth. Overall, this work has developed an approach to rapidly delineate members of TF families that are potentially linked to physiological processes through their activity on promoter target sequences. This approach is anticipated to accelerate decision making to carry out functional analyses such as stable transformation in *Arabidopsis* or spruce and more detailed analyses of the DNA-binding region for a specific TF, as recently reported by Bomal *et al.* (2014).

## Supplementary material

Supplementary data are available at *JXB* online.

Supplementary Fig. S1. Clustering of gene expression using SOTA analysis.

Supplementary Table S1. List of gene-specific primers used for quantitative PCR analysis.

Supplementary Table S2. List of primers used for identification and cloning of candidate gene 5′-genomic sequences using the GenomeWalker kit.

Supplementary Table S3. Comparison of transcript levels of a set of 15 genes on day 1 and day 2.

Supplementary Table S4. Gene identifiers, GenBank accessions, cDNA insert size, and functional annotations.

Supplementary Table S5. Pfam functional annotations for genes studied.

Supplementary Table S6. Transcript accumulation, statistical analyses, and clustering results.

Supplementary Table S7. Determination of the most stable reference genes and calculation of gene expression normalization factor using geNorm.

Supplementary Data
