# Supplementary material for: Large-scale screening of transcription factor–promoter interactions in spruce reveals a transcriptional network involved in vascular development
Source: J Exp Bot. 2014 Apr 8;65(9):2319–33. doi: 10.1093/jxb/eru116 (PMC4036505; doi:10.1093/jxb/eru116)
Supplement: Supplementary Data [file supp_65_9_2319__index.html]

Large-scale screening of transcription factor–promoter interactions in spruce reveals a transcriptional network involved in vascular development — Supplementary Data 

# Large-scale screening of transcription factor–promoter interactions in spruce reveals a transcriptional network involved in vascular development

## Supplementary Data

Data files

**Files in this Data Supplement:**

- Supplementary Data - Supplementary Data
- Supplementary Data - Supplementary Data
