# Supplementary material for: Large-scale screening of transcription factor–promoter interactions in spruce reveals a transcriptional network involved in vascular development
Source: J Exp Bot. 2014 Apr 8;65(9):2319–33. doi: 10.1093/jxb/eru116 (PMC4036505; doi:10.1093/jxb/eru116)
Supplement: Supplementary Data [file supp_eru116_Duval_et_al_FigS1_final.pdf]

Large-scale screening of transcription factor – promoter interactions in spruce revealed a transcriptional network involved in vascular development

Isabelle Duval, Denis Lachance, Isabelle Giguère, Claude Bomal, Marie-Josée Morency, Gervais Pelletier, Brian Boyle, John J. MacKay, Armand Séguin

Fig. S1. Clustering of gene expression using Self-Organizing Tree Algorithm (SOTA) analysis.

Fig. S1

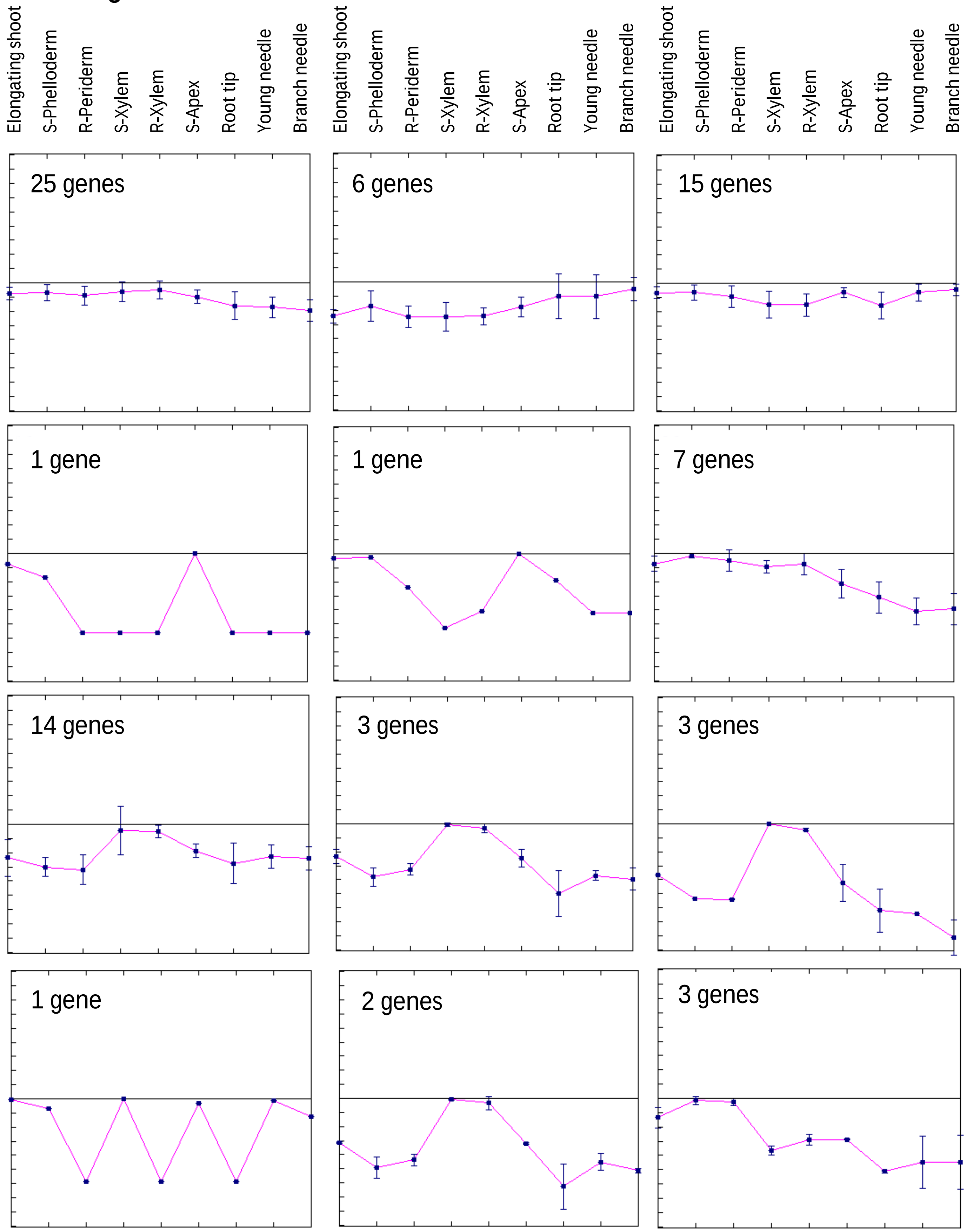

Elongating shoot
S-Phelloderm
R-Periderm
S-Xylem
R-Xylem
S-Apex
Root tip
Young needle
Branch needle
25 genes
6 genes
15 genes
1 gene
1 gene
7 genes
14 genes
3 genes
3 genes
1 gene
2 genes
3 genes
